# Insight into redox regulation of apoptosis in cancer cells with multiparametric live-cell microscopy

**DOI:** 10.1038/s41598-022-08509-1

**Published:** 2022-03-16

**Authors:** Marina V. Shirmanova, Alena I. Gavrina, Tatiana F. Kovaleva, Varvara V. Dudenkova, Ekaterina E. Zelenova, Vladislav I. Shcheslavskiy, Artem M. Mozherov, Ludmila B. Snopova, Konstantin A. Lukyanov, Elena V. Zagaynova

**Affiliations:** 1 Privolzhsky Research Medical University, Minin and Pozharsky Sq. 10/1, 603005 Nizhny Novgorod, Russia; 2 National Medical Research Radiological Centre of the Ministry of Health of the Russian Federation, 2nd Botkinsky proezd, 3, Moscow, Russia 125284; 3 Becker&Hickl GmbH, Nunsdorfer Ring 7-9, 12277 Berlin, Germany; 4 Skolkovo Institute of Science and Technology, Bolshoy Boulevard 30, bld. 1, Moscow, Russia 121205; 5 Lobachevsky State University of Nizhny Novgorod, Gagarin Avenue 23, Nizhny Novgorod, Russia 603950

**Keywords:** Biophysics, Cancer

## Abstract

Cellular redox status and the level of reactive oxygen species (ROS) are important regulators of apoptotic potential, playing a crucial role in the growth of cancer cell and their resistance to apoptosis. However, the relationships between the redox status and ROS production during apoptosis remain poorly explored. In this study, we present an investigation on the correlations between the production of ROS, the redox ratio FAD/NAD(P)H, the proportions of the reduced nicotinamide cofactors NADH and NADPH, and caspase-3 activity in cancer cells at the level of individual cells. Two-photon excitation fluorescence lifetime imaging microscopy (FLIM) was applied to monitor simultaneously apoptosis using the genetically encoded sensor of caspase-3, mKate2-DEVD-iRFP, and the autofluorescence of redox cofactors in colorectal cancer cells upon stimulation of apoptosis with staurosporine, cisplatin or hydrogen peroxide. We found that, irrespective of the apoptotic stimulus used, ROS accumulation correlated well with both the elevated pool of mitochondrial, enzyme-bound NADH and caspase-3 activation. Meanwhile, a shift in the contribution of bound NADH could develop independently of the apoptosis, and this was observed in the case of cisplatin. An increase in the proportion of bound NADPH was detected only in staurosporine-treated cells, this likely being associated with a high level of ROS production and their resulting detoxification. The results of the study favor the discovery of new therapeutic strategies based on manipulation of the cellular redox balance, which could help improve the anti-tumor activity of drugs and overcome apoptotic resistance.

## Introduction

Apoptosis, or programmed cell death, is a highly ordered and controlled biological process that plays a very important role in many diseases, including cancer.

Reactive oxygen species (ROS) are involved in the initiation and regulation of the intrinsic (mitochondrial) apoptotic cascade. Increases of ROS concentrations during apoptosis have been documented for many types of cancer cells and for different apoptotic stimuli, including chemotherapy and radiation therapy^1^. The apparent role of ROS is to trigger the release of cytochrome *c* from mitochondria to the cytoplasm, where it induces formation of the apoptosome and subsequent caspase activation. At the same time, the activated caspases can increase ROS levels due to their effects on the mitochondrial respiratory complexes^2^. However, the origin of the ROS involved in apoptotic death of cancer cells is still debatable.

The major sources of ROS production in cells are the mitochondrial electron transport chain (ETC) and the NADPH oxidases. Oxidative phosphorylation (OXPHOS) in mitochondria involves four electron-transporting complexes and ATP synthase that direct electrons derived from the initial oxidation of NADH and FADH_2_ along a multistep pathway that culminates in protons being pumped outside of the mitochondria. In mitochondria, it is mainly complexes I and III that generate superoxide (O_2_·^–^) through the univalent reduction of molecular oxygen as a result of electron leakage during respiration. NADPH oxidase, a family of highly regulated membrane enzymes, oxidizes NADPH to generate superoxide. Because of defective mitochondrial function and overexpression of NADPH oxidase, cancer cells often have elevated levels of ROS and higher levels of ROS scavenging enzymes compared to normal cells^3,4^.

The nicotinamide adenine dinucleotide (NAD^+^/NADH) and nicotinamide adenine dinucleotide phosphate (NADP^+^/NADPH) redox couples are the major determinants of the redox state in the cell. The NAD^+^/NADH couple is involved in glycolysis, the tricarboxylic acid (TCA) cycle and mitochondrial respiration, the last of these being associated with the formation of ROS. NADP^+^/NADPH participates in biosynthetic pathways and in antioxidant defense, but it may also serve as a cofactor in free radical oxidation reactions^5^. Both cofactors are important in control of the balance between the production of ROS and their neutralization. Tumor cells produce greater amounts of the reduced forms of the nicotinamide cofactors, NADH and NADPH in comparison with normal cells^2^.

The reduced forms of these cofactors (denoted NAD(P)H) produce autofluorescence in the blue spectral range, the lifetime of which is an established metric of cellular metabolism. Numerous studies have shown that fluorescence lifetime imaging microscopy (FLIM) of NAD(P)H can detect changes in flux through glycolysis, the TCA cycle and the mitochondrial ETC^6,7^. In addition, as shown by Blacker et al., FLIM can differentiate NADH from NADPH, as these two forms have different fluorescence lifetimes in their protein-bound state^8^. The ratio of the fluorescence intensities of NAD(P)H and oxidized flavin adenine dinucleotide (FAD) is an additional useful metric of the cellular redox state. The fluorescence lifetime of NAD(P)H and the intensity-based redox ratio change upon the induction of apoptosis^9–11^, however the molecular processes responsible for these changes have yet to be identified.

It is evident that ROS generation, and the NADH and NADPH pools are interconnected, but their relationship to apoptosis and the dynamics of the changes during apoptosis execution remain poorly investigated. Our study was aimed at finding correlations between ROS, the redox ratio of FAD/NADH, the proportions of NADH and NADPH and caspase-3 activity at the individual cell level, using multiparametric time-lapse microscopy. The activation of caspase-3, a key effector caspase, was detected using the new, far-red, genetically encoded sensor mKate2-DEVD-iRFP, stably expressed in colorectal cancer cells^12^. To induce apoptosis, we used staurosporine (STS), the chemotherapeutic drug cisplatin or hydrogen peroxide. All the parameters were correlated against the intracellular production of ROS. The data on NAD(P)H fluorescence lifetimes after staurosporine treatment were then validated on mouse tumors in vivo.

## Results

### Caspase-3 activity in vitro

We monitored caspase-3 activity as an increase in the donor fluorescence lifetime (or reduction of the efficiency of Fortser Resonance Energy Transfer (FRET)) using the genetically encoded sensor mKate2-DEVD-iRFP.

The fluorescence lifetime of mKate2 in the control cells was 1.53 ± 0.10 ns.

**Figure 1:**
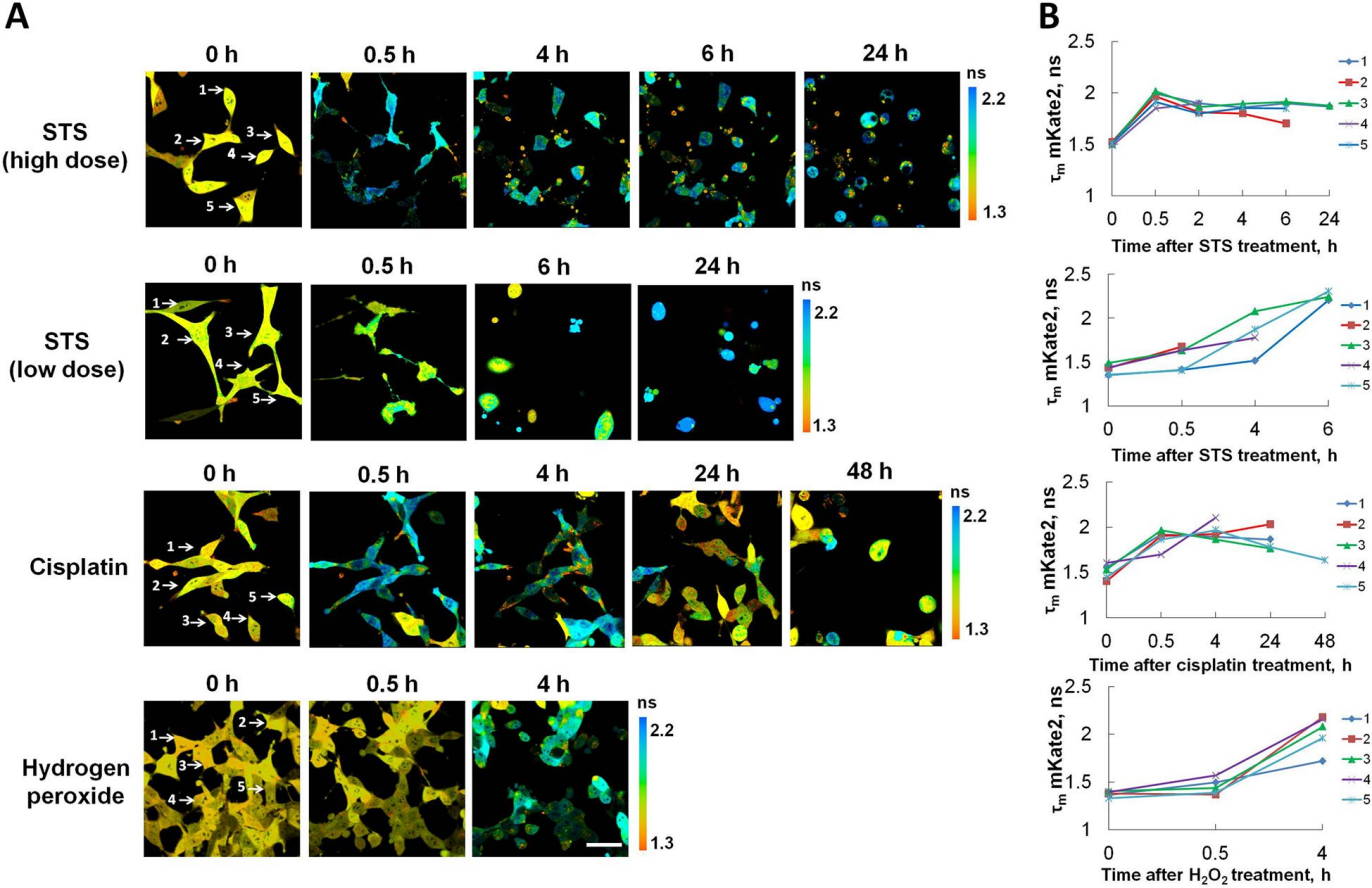
Caspase-3 activity in CT26 cancer cells stably expressing the genetically encoded FRET-based sensor, mKate2-DEVD-iRFP, upon induction of apoptosis. (**A**) Time-lapse FLIM images of the donor mKate2 before (0 h) and after treatment with staurosporine (STS), cisplatin or hydrogen peroxide. Image size is 213 × 213 μm. Scale bar: 50 μm. (**B**) Fluorescence lifetime of mKate2 in the individual cells shown in (**A**). Individual cells in (**A**) are numbered.

At high concentration of STS (5 µM), the onset of caspase-3 activation occurred early and could be detected after 0.5 h in most of the cells (81%); the fluorescence lifetime of the mKate2 increased up to 1.86 ± 0.08 ns. These changes were accompanied by cell shrinkage and membrane blabbing, the defined morphological features of apoptosis, followed by the formation of apoptotic bodies (Fig. 1). At the late apoptotic stages (time-points > 4 h), tracking the fluorescence of the caspase-3 sensor was not feasible due to inadequate mKate2 fluorescence lifetimes (1.68–1.79 ns) in the cytoplasm of the dying cells. Lowering the STS concentration to 0.1 µM delayed the activation of caspase-3 and reduced the percentage of cells undergoing caspase-3 activation; at 6 h, the fluorescence lifetime was 1.78 ± 0.32 ns in a total cell population that included 64% apoptotic cells; at 24 h all cells displayed an increased mKate2 fluorescence lifetime (2.19 ± 0.31 ns).

Treatment with cisplatin (2.2 µM) led to an increase in the fluorescence lifetime of mKate2 in the bulk of cells (75%) starting from 0.5 h (1.88 ± 0.13 ns), which did not change further, up to 4 h. In 24–48 h the cell population consisted of both apoptotic (2.04 ± 0.15 ns) and surviving (1.59 ± 0.11 ns) cells in almost equal proportions. Tracking the apoptotic process in individual cells allowed us to conclude that the appearance of the cells with shorter fluorescence lifetimes of the donor on prolonged exposure resulted from the proliferation of a small sub-population of cells resistant to apoptosis (Fig. 1).

Exposure to H_2_O_2_ (1 mM) led to a pronounced increase in caspase-3 activity, peaking at 4 h; the mKate2 fluorescence lifetime was 1.90 ± 0.16 ns and the fraction of apoptotic cells was 77% (Fig. 1).

### Redox ratio FAD/NAD(P)H in vitro

**Figure 2:**
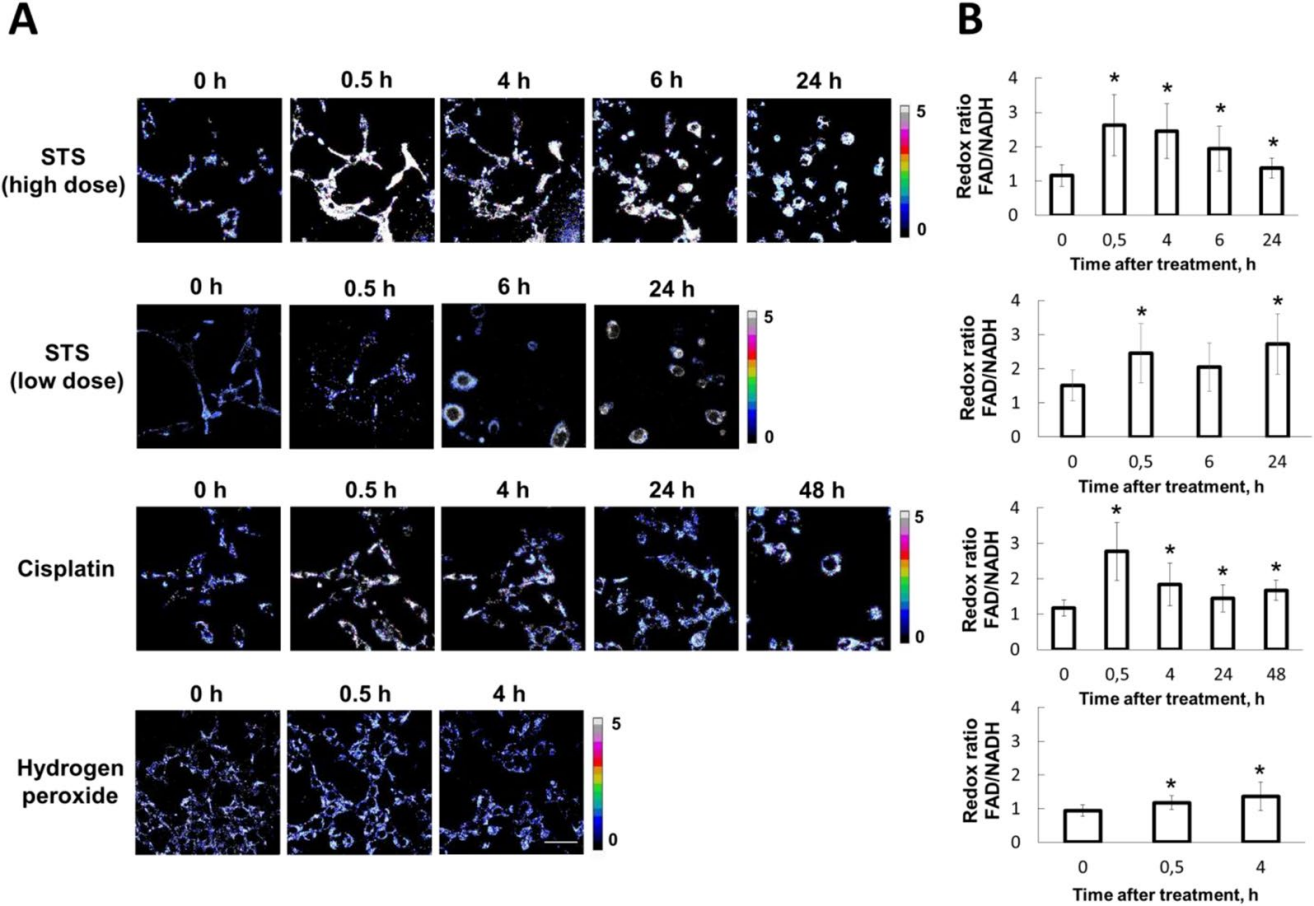
Optical redox ratio FAD/NADH in CT26 cancer cells stably expressing the genetically encoded FRET-based sensor, mKate2-DEVD-iRFP, upon induction of apoptosis. (**A**) Time-lapse imaging of the ratio FAD/NADH before (0 h) and after treatment with staurosporine (STS), cisplatin or hydrogen peroxide. Image size is 213 × 213 μm. Scale bar: 50 μm. (**B**) Quantification of the optical redox ratio FAD/NADH in the cells. Mean ± SD, n = 20–50 cells. *p ≤ 0.05 with control (0 h). Images were acquired from the same fields of view as in Fig. 1.

Following induction of apoptosis with STS, we found that the redox ratio FAD/NAD(P)H significantly increased from ~ 1.1 to ~ 2.6 (p = 0.000) in all cells at 0.5 h and then remained at this increased level until 24 h (Fig. 2).

In cisplatin-treated cells, an increase in the redox ratio (from ~ 1.1 to ~ 2.8, p = 0.000) was observed at 0.5 h followed by a gradual decrease during the next 24 h. A separate analysis of the redox ratio in apoptotic and non-apoptotic cells revealed no differences between these two groups of cells, suggesting that elevated oxidative status is a non-specific response to cisplatin.

In cells exposed to H_2_O_2_ the redox ratio gradually increased from ~ 0.9 to ~ 1.3 (p = 0.0001) over a period 4 h.

Therefore, the redox ratio measurements following treatment of cancer cells with staurosporine, cisplatin or H_2_O_2_ showed a shift to a more oxidative status for all the agents used.

### FLIM of NADH and NADPH in vitro

**Figure 3:**
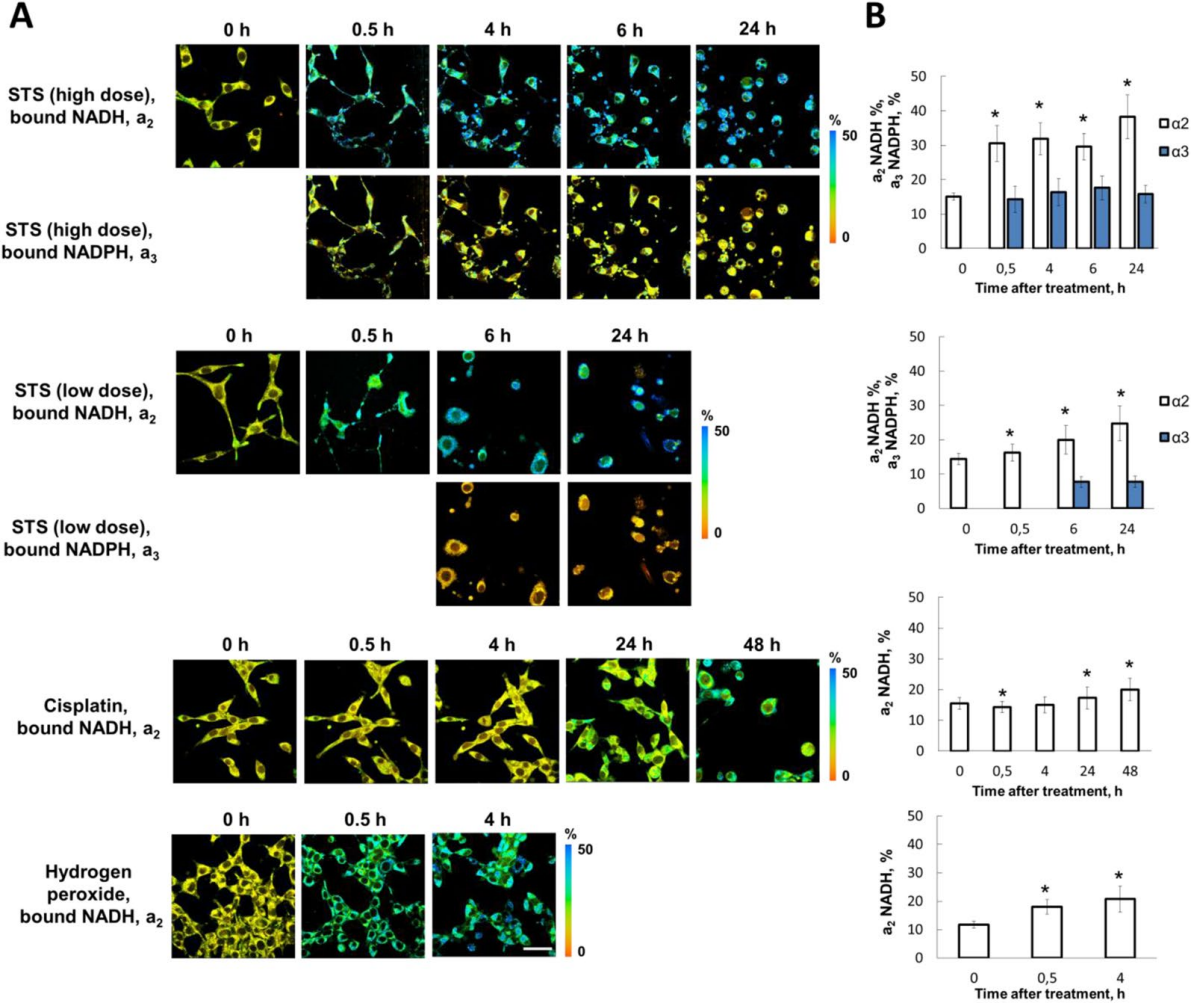
FLIM of NAD(P)H in CT26 cancer cells stably expressing the genetically encoded FRET-based sensor, mKate2-DEVD-iRFP, upon induction of apoptosis. (**A**) Time-lapse images of the relative contributions of bound NADH (a_2_) and bound NADPH (a_3_) before (0 h) and after treatment with staurosporine (STS), cisplatin or hydrogen peroxide. Image size is 213 × 213 μm. Scale bar: 50 μm. (**B**) Quantification of the relative contributions of the bound NADH (a_2_) and bound NADPH (a_3_) in the cells. Mean ± SD, n = 20–50 cells. *p ≤ 0.05 with control (0 h). NAD(P)H fluorescence decay curves for STS were processed with three-exponential fitting; and for cisplatin and hydrogen peroxide—with bi-exponential fitting. Images were acquired from the same fields of view as in Figs. 1 and 2.

Firstly, the fluorescence decay curves for NAD(P)H were fitted with a classical bi-exponential model, where the short component was attributed to free NAD(P)H and the long component—to protein-bound NAD(P)H (Fig. 3). The fluorescence lifetimes of the free (τ_1_) and protein-bound (τ_2_) NAD(P)H before apoptosis induction were 0.47 ± 0.06 ns and 2.85 ± 0.19 ns, respectively, which is consistent with the typical values described in the literature^5,8,13,14^. STS treatment led to an increase in τ_2_-NAD(P)H, and this increase was significantly more pronounced with higher dose of STS: 5.55 ± 0.76 ns at 0.5 h, p = 0.0000. For comparison, at lower STS doses, τ_2_-NAD(P)H was 3.01 ± 0.19 ns, p = 0.0008 at 6 h. However, after treatment with cisplatin or H_2_O_2_ τ_2_-NAD(P)H did not change (Table S1).

It is known that the fluorescence lifetime of bound NAD(P)H in cells primarily depends on the type of NADH- and NADPH-binding enzymes. Increased τ_2_-NAD(P)H (typically > 3.5 ns) indicates an increased contribution of NADPH, that has a fluorescence lifetime of 4.4 ns^8^. In line with this, we processed the FLIM data with typical fluorescence lifetimes (control, cisplatin and H_2_O_2_) using bi-exponential fitting and attributed the long-lifetime fraction to protein-bound NADH, while the FLIM data with increased τ_2_ (STS) were processed using three-exponential fitting, where the relative amplitude a_2_ was attributed to protein-bound NADH and the relative amplitude a_3_ was attributed to the protein-bound NADPH fraction.

The relative contribution of bound NADH (a_2_) in untreated cells was ~ 14%. Treatment of cells with any of the agents resulted in an elevation of a_2_-NADH (Fig. 3).

After adding STS, a_2_-NADH gradually increased starting from 0.5 h and reached maximum values after 24 h: ~ 38% at 5 µM and ~ 25% at 0.1 µM concentrations. The relative contribution of bound NADPH (a_3_) was ~ 14% at 0.5 h after treatment with 5 µM STS and remained at the elevated level up to 24 h. With 0.1 µM STS the value of a_3_-NADPH was ~ 8% starting from 6 h.

An increase in a_2_-NADH (17.36 ± 1.87, p = 0.007) was detected from 24 h after treatment with cisplatin. At 48 h, a_2_-NADH increased to 20.04 ± 3.67% (p = 0.000). No significant difference in a_2_-NADH was observed between the apoptotic and non-apoptotic cells.

H_2_O_2_ treatment was accompanied by an increase of a_2_-NADH from 0.5 h (18.08 ± 2.55%, p = 0.000). During the next 4 h a_2_-NADH continued to increase to 20.77 ± 4.60% (p = 0.000).

Comparison of the dynamics of the optical metabolic readouts and apoptotic process shows that metabolic changes preceded the activation of caspase-3 induced by the STS and H_2_O_2_ treatments. In the case of cisplatin, the observed metabolic changes were not associated with induction of apoptosis and evolved equally late in all cells.

### Simultaneous FLIM of caspase-3 and NAD(P)H in tumors in vivo

**Figure 4:**
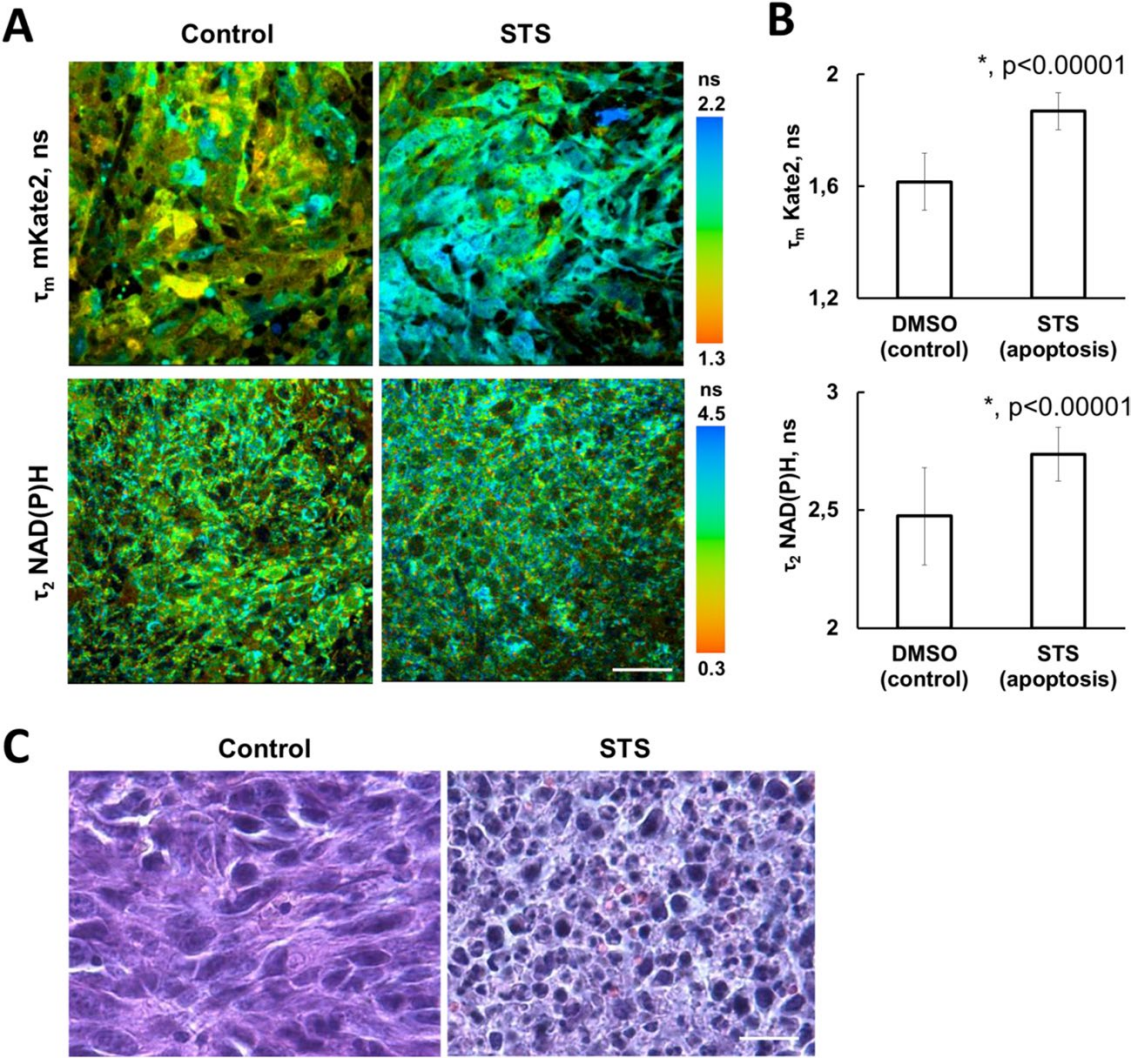
Simultaneous in vivo FLIM of mKate2 and NAD(P)H in CT26 tumors expressing the genetically encoded FRET-based sensor of caspase-3 activity, mKate2-DEVD-iRFP. (**A**) Representative FLIM images of mKate2 and NAD(P)H before (control) and after treatment with staurosporine (STS). Image size is 213 × 213 μm. Scale bar: 100 μm. (**B**) Quantification of the fluorescence lifetime of mKate2 and the fluorescence lifetime of bound NADH (τ_2_) in the tumor cells. Mean ± SD, n = 20–50 cells. *p ≤ 0.00001 with control. The NAD(P)H fluorescence decay curves were processed with bi-exponential fitting. (**C**) Histopathological analysis of the control and STS-treated tumors. H&E staining. Scale bar: 50 μm.

To test whether similar metabolic changes occur during apoptosis in tumors in vivo, we performed experiments on mice with CT26 tumors, expressing the mKate2-DEVD-iRFP sensor (Fig. 4). Upon intratumoral injection of STS, areas with activated caspase-3 were found in the tumors, this being verified by histopathology. As expected, apoptotic cells in STS-treated tumors had a longer fluorescence lifetime of mKate2 than the cells of control tumors, ~ 1.87 ns vs. ~ 1.63 ns. FLIM of NAD(P)H revealed a statistically significant increase of τ_2_-NAD(P)H in the STS-treated tumors from ~ 2.47 to ~ 2.74 ns, both in the apoptotic and non-apoptotic cells. Increase in τ_2_-NAD(P)H in the non-apoptotic cells conforms with the in vitro data that showed no correlation between τ_2_-NAD(P)H and caspase-3 activation (*r* = 0.266). Analysis of the relative contributions of free and bound NAD(P)H did not display any changes after the treatment. Therefore, the in vivo study revealed a similar trend in the metabolic rearrangements accompanying the apoptotic process in terms of NAD(P)H fluorescence lifetime but, in general, the metabolic changes were less apparent compared with cancer cells in a cell culture.

### Production of ROS

**Figure 5:**
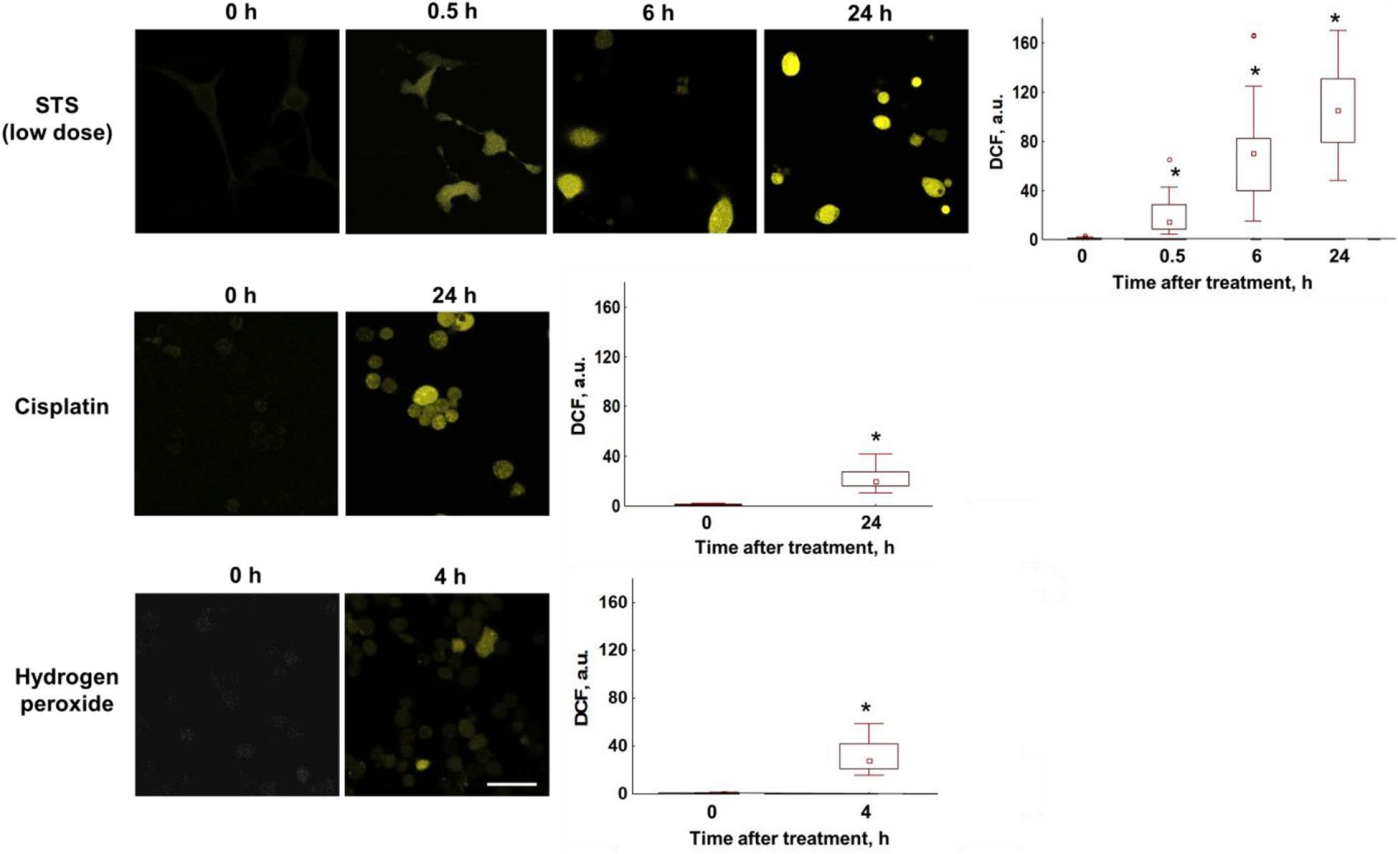
ROS level in CT26 cancer cells stably expressing the genetically encoded FRET-based sensor, mKate2-DEVD-iRFP, upon induction of apoptosis. Left: Time-lapse microscopic images of cells stained with the ROS-sensitive probe H_2_DCFDA, before (0 h) and after treatment with staurosporine (STS), cisplatin or hydrogen peroxide. Image size is 213 × 213 μm. Scale bar: 50 μm. Right: Quantification of fluorescence intensity of DCF in the cells. Box-and-Whisker plots display the median, 25th and 75th percentiles, minimum and maximum. n = 20–50 cells. *p ≤ 0.05 with control (0 h).

An analysis of the fluorescence intensity of the DCF dye showed its gradual increase starting from 0.5 h after adding STS (0.1 µM), when metabolic changes could be detected but activity of caspase-3 was not. The maximum increase (104-fold) in intensity was observed at 24 h. After treatment with H_2_O_2_ and cisplatin a moderate increase in the DCF signal was observed in 4 h and 24 h, respectively (Fig. 5). Staining with the redox-insensitive dye CDCF showed no changes in its fluorescence intensity after treatment of cells with any of three agents (Fig. S1), which indicated that factors other than oxidants did not contribute to the DCF signal.

### Correlation analysis

**Figure 6:**
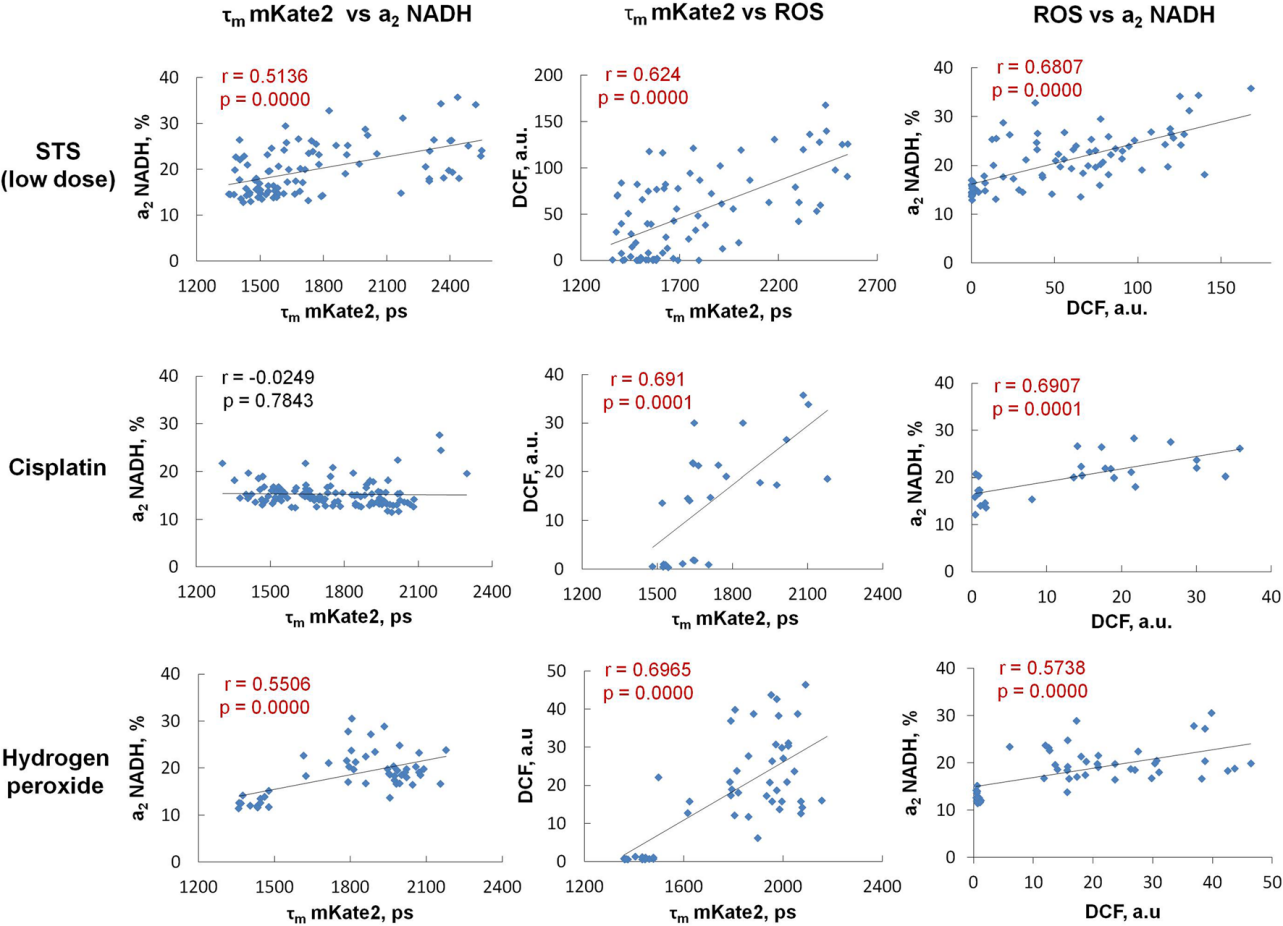
Correlations between caspase-3 activity (τ_m_ mKate2), the protein-bound NADH fraction (a_2_-NADH) and the ROS levels (DCF) in cancer cells upon induction of apoptosis. Blue dots are the measurements for individual cells. Pearson’s correlation coefficient and p-value are indicated on each plot. The solid line represents the regression line.

We investigated the correlations between caspase-3 activity (mKate2 fluorescence lifetime), metabolic state (redox ratio FAD/NAD(P)H, and NADH and NADPH contributions) and ROS levels at the single cell level for each of the agents used to induce apoptosis (Fig. 6, Table S2).

Pearson’s correlation test showed a good positive correlation between caspase-3 activity and intracellular ROS, as well as between ROS and the bound NADH fraction for all agents, which indicates that: (1) ROS are involved in caspase-3-mediated apoptotic pathways and (2) ROS production is connected to the elevated mitochondrial NADH pool. A moderate positive relationship was found between caspase-3 activity and the fraction of bound NADH for the STS and H_2_O_2_ treatments, suggesting that the observed metabolic changes are associated with apoptosis, while cisplatin induced similar metabolic alterations irrespective of any apoptotic process. Furthermore, the FAD/NAD(P)H redox ratio showed only weak correlations with caspase-3 activation and the level of ROS, irrespective of the type of stimuli. NADPH, detected only in the case of STS, did not correlate with any of the studied parameters. Along with the redox ratio, this observation implies high cell-to-cell variability in the regulation of redox status during apoptosis.

## Discussion

**Figure 7:**
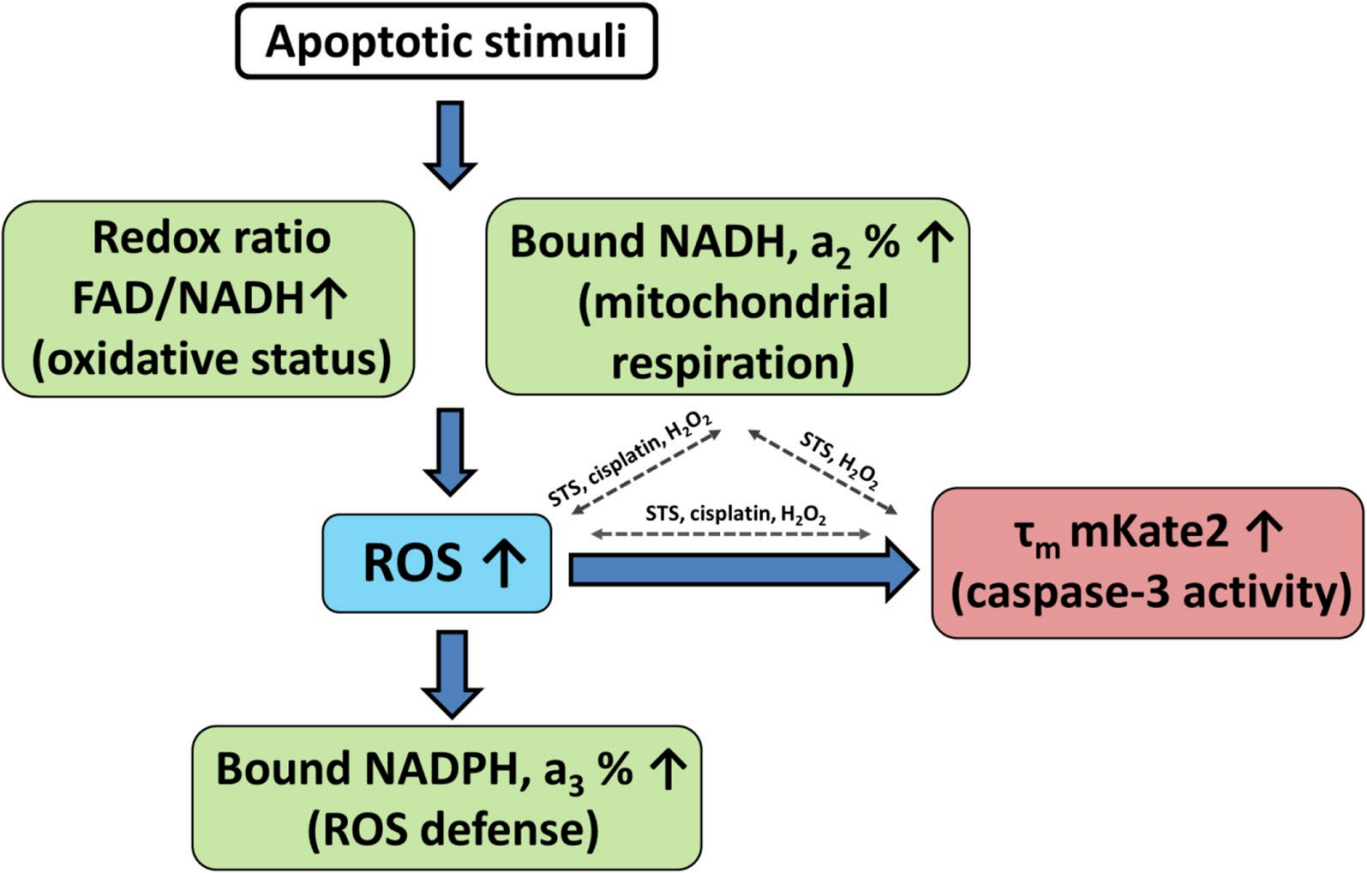
Schematic of the associations between the redox ratio FAD/NADH, the metabolic cofactors NADH and NADPH, the production of ROS, and caspase-3 activity in cancer cells. Blue arrows show the order of events. Gray dashed arrows show correlations between the events revealed for the specific apoptotic stimuli (STS, cisplatin or H_2_O_2_).

This study presents an insight into the changes in the redox and energy metabolism of cancer cells during apoptosis induced by different agents—STS, cisplatin and H_2_O_2_. In our previous study, using FLIM, we observed an increase in the optical redox ratio FAD/NAD(P)H and a decrease in the relative contribution of free NAD(P)H in cancer cells shortly after exposure to STS that pointed to a shift toward oxidative metabolism^14^. In addition, we registered the significantly elongated fluorescence lifetime of protein-bound NAD(P)H, τ_2_. In the current study we have tried to analyze the NAD(P)H fluorescence decay parameters to identify the NADPH pool during STS-induced apoptosis and have investigated whether similar metabolic alterations accompany apoptosis induced by other stimuli. For the first time, multiparameter fluorescence imaging of caspase-3 activity, of the optical redox ratio, of the metabolic cofactors NADH and NADPH and of intracellular ROS in individual cells has been performed. Cell death, an increase in the proportion of protein-bound NADH and the production of ROS all showed a strong correlation in each case. A schematic illustration of the associations between the redox ratio of FAD/NADH, the levels of the metabolic cofactors NADH and NADPH, the production of ROS, and of caspase-3 activity in cancer cells upon apoptotic stimulation is shown in Fig. 7.

The present research was conducted using a model consisting of monolayer cells grown on gridded culture dishes as this allowed us to follow the responses of individual cells to apoptotic stimuli over long time periods. Given the heterogeneous dynamics of the apoptotic process in a cell population, monitoring of several parameters simultaneously in the same cells was crucial to exploring the metabolic aspects of apoptosis. Of course, all cell culture models have limitations on their representativeness of physiological conditions. In solid tumors, the natural microenvironment including the host’s immune system, the heterogeneous distribution of oxygen and nutrients, and a complex network of intercellular interactions, is believed to have a profound regulatory effect on the apoptotic program^15^, which is, unfortunately, difficult to study in vivo. However, our preliminary results in vivo on the simultaneous imaging of NAD(P)H and caspase-3 activity in mouse tumors, while showing a tendency toward metabolic rearrangements similar to those in our in vitro model, imply stronger compensatory mechanisms to prevent the altering of the redox homeostasis.

We used three agents that induce apoptosis through the mitochondrial pathway. Staurosporine (STS), a broad-spectrum kinase inhibitor, is the classical inducer of the intrinsic (mitochondrial) apoptotic pathway. Cisplatin is a well-known anticancer chemotherapeutic drug that damages DNA through the formation of platinum–DNA adducts. Such DNA-damage signals activate downstream signaling cascades involving p53, MAPK, and p73 that ultimately induce apoptosis. Cisplatin’s induction of apoptosis as a result of mitochondrial damage has also been described, although this is a rather less appreciated mechanism of its action^16,17^. Exogenous H_2_O_2_, applied at low concentrations, induces apoptotic cell death in a caspase-dependent manner, presumably via rapid mitochondrial changes^17–19^.

To detect apoptosis we used the genetically encoded FRET-based sensor of caspase-3 activity, mKate2-DEVD-iRFP^12^. Upon induction of apoptosis with any of the agents, we observed, in the cell population, a high variability of mKate2 fluorescence lifetimes that covered a whole range of values from fully bound to the acceptor, to fully unbound, indicating different degree of caspase-3 activity.

Intracellular ROS are tightly linked to the activation of the mitochondrial apoptotic pathway, where they are produced as a result of leakage from the respiratory ETC. A large body of evidence has shown that during staurosporine-induced apoptotic death, generation of ROS occurs^16,20,21^. Induction by cisplatin of ROS, most of which are generated by the mitochondria, was detected in different cell lines and it correlated with their sensitivity to the drug^22,23^. It has been suggested that cisplatin-induced ROS generation occurs as a consequence of its direct effect on mitochondrial DNA, resulting in impairment of ETC protein synthesis^24^ and enhanced mitochondrial biogenesis^25^. In H_2_O_2_-induced apoptosis, the release of mitochondrially generated ROS has also been identified^26^. Comparison of the ROS levels in our study shows that staurosporine, even at low dosage (0.1 µM) induced a 4.75-fold higher ROS generation than did cisplatin or H_2_O_2_.

We found that strong associations exist between caspase-3 activation and the generation of ROS at the single-cell level, irrespective of the apoptotic stimuli. Previously, Ricci et al. using staurosporine, actinomycin D, or UV as apoptosis inducers, suggested that the production of ROS during apoptosis can be caspase dependent^27^. They showed that caspase-3 acts on the permeabilized mitochondria to disrupt transmembrane potential and respiration, and induces ROS production via its action on complexes I and II. On the other hand, results by Brentnall et al. suggested that caspase-3 inhibits ROS production, while caspase-7 may be required for ROS accumulation during intrinsic apoptosis^28^. In the case of H_2_O_2_-induced apoptosis, early mitochondrial events, such as the generation of permeability transition pores, a drastic decrease in the mitochondrial transmembrane potential, and the release of mitochondrial ROS, precede the activation of caspase-3^26^. In our study, monitoring of caspase-3 activation in parallel with ROS upon staurosporine treatment showed that ROS are already being produced at 0.5 h, before caspase-3 has actually been activated, which indicates that ROS play a role at the stage of initiation of apoptosis. However, it can not be ruled out that activated caspase-3 can also contribute to the generation of ROS.

The ratio of the endogenous fluorescence of the metabolic coenzymes NADH and FAD is an established indicator of cellular redox status. It has previously been verified that the intensity-based redox ratios FAD/(NADH + FAD) or FAD/NADH are highly correlated with biochemical assessments of the NAD^+^/(NADH + NAD^+^) ratio using mass spectrometry^29^ or indicated by assessing cellular oxygen consumption^30^. We observed an increase in the redox ratio FAD/NADH in all treated cells, irrespective of apoptosis development or ROS generation. Paralleling our study, increases in the redox ratio FAD/(NADH + FAD) and in ROS were detected by Podsednik et al. in breast cancer cells following the addition of H_2_O_2_^31^. We suggest that such a non-specific shift to a more oxidative state could be caused by elevated oxidative stress and/or DNA damage induced by the treatments.

An increased redox ratio of FAD/NADH and ROS production are consistent with the observed elevation in the bound NADH fraction (a_2_). In the context of the apoptotic process, a possible explanation for the shift toward increased NADH-a_2_ may be the stimulation of mitochondrial respiration. Recently, it was proposed that the activity of the ETC is controlled by the phosphorylation of cytochrome *c*. Dephosphorylation of cytochrome *c* results in maximal ETC flux, hyperpolarization of the membrane potential ΔΨ_m_, and ROS production, leading to apoptotic cell death. This regulatory mechanism is, at least partly, involved in both H_2_O_2_- and STS-induced apoptosis^32^ and explains our observation that caspase-3 activity correlates with NADH-a_2_ upon treatment with H_2_O_2_ or STS. In the case of cisplatin, the increase of NADH-a_2_ was a non-specific response, observed in all cells. This may also be due to increased OXPHOS, as it is considered a beneficial, adaptive response to DNA damage^33^. Previously, we have demonstrated that increased NADH-a_2_ and FAD/NADH ratios are common responses of cancer cells to chemotherapeutic drugs, including cisplatin^34^.

We did not observe any significant fluctuations in the bound NAD(P)H lifetime (τ_2_) in cells treated with cisplatin or H_2_O_2_, while treatment with STS resulted in an increase in the lifetime, both in vitro and in vivo, which we attributed to NADPH. The role of NADPH in apoptosis is not fully determined as yet. On the one hand, it is involved in ROS detoxification as the donor of reductive potential to glutathione and thioredoxins. On the other hand, NADPH plays an important role as a source for ROS being a cofactor of the NADPH oxidases (NOX)^35–40^. In our study, ROS production preceded NADPH accumulation and these two processes only weakly correlated with each other, which collectively suggest that, during STS-induced apoptosis, NADPH is involved in ROS detoxification rather than in ROS production.

A limitation of our study is that we used only DCF for the assessment of the ROS level. Although the oxidation-sensitive H_2_DCFDA probe is one of the most widely used for detection of ROS, it is not specific for any particular oxidant. Moreover, due to the complex intracellular redox chemistry of the probe, some artifacts cannot be ruled out, e.g. DCFH oxidation by cytochrome *c* or interaction of the intermediate products with oxygen, forming additional ROS. Other factors that have to be taken into account are the presence of peroxynitrite or redox-active metals (e.g. Fe^2+^)^41,42^. However, given the good correlation between DCF fluorescence, the mitochondrial NADH pool and caspase-3 activity, plus supporting literature data about ROS in apoptosis, we suggest that factors other than the ROS had only minor effects on the DCF signal. Additional biochemical tests and control experiments are required to identify the type and precise localization of the ROS generated during apoptosis.

## Conclusions

Insight into the molecular mechanism of apoptosis opens new avenues for the development of diagnostic, prognostic, and therapeutic tools for the management of cancer. In this study, cellular metabolism, redox homeostasis and the dynamics of the apoptotic process were monitored in cancer cells in vitro using live-cell fluorescence microscopy. We showed, for the first time, that caspase-3 activation is accompanied by an increase in the proportion of bound NADH, attributed to mitochondrial OXPHOS, and to elevation of the level of ROS. Furthermore, an extremely high ROS production promoted an increase in the proportion of bound NADPH up to a level detectable by FLIM. Altogether, these data suggest that any variations in the NADH and NADPH fluorescence decay parameters are specific to the redox state changes and show the potential for “label-free” estimation of metabolic rearrangements that may occur in cancer cells during therapeutic interventions. It is possible that the contributions of metabolic/redox intermediates and their relevance to apoptosis would vary if assessed in tumors, as a consequence of more complex metabolic and genetic alterations and the specific microenvironment. Further studies on tumors will be necessary to validate the proposed relationships in the cellular redox system.

## Materials and methods

All methods were carried out in accordance with relevant guidelines and regulations.

### Cell culture

A CT26 (murine colon carcinoma) cell line, stably expressing the genetically encoded FRET-based sensor for caspase-3 activity, mKate2-DEVD-iRFP^12^, was used. The cells were cultured in DMEM supplemented with 10% fetal calf serum (GE Healthcare Life Sciences, USA), 2 mM glutamine (PanEco, Russia), 10 U/mL penicillin, and 10 mg/mL streptomycin (PanEco, Russia) and maintained at 37 °C in a humidified 5% CO_2_ incubator.

For fluorescence microscopy, the cells were seeded (8 × 10^4^ in 2 mL) into glass-bottomed grid-50 dishes (Ibidi, Germany) in DMEM without phenol red (Life Technologies). During time-lapse imaging, the cells were maintained at 37 °C and 5% CO_2_. Images of the same cells were collected before treatment (control) and at different time-points (30–48 h) afterwards. Imaging was performed in the following order: fluorescence intensities of NAD(P)H and FAD → FLIM of mKate2 → FLIM of NAD(P)H. In a separate experiment, the cells were stained for ROS at the selected time-points after mKate2 and NAD(P)H FLIM, and imaged in the same fields of view.

Apoptosis was induced with 0.1 µM or 5 µM staurosporine (Enzo Life Sciences, USA), 2.2 µM cisplatin (Teva, Israel) or 1 mM hydrogen peroxide. The dose of cisplatin corresponded to the half-inhibitory concentration IC50, as determined previously by an MTT-assay.

For the assessment of morphological changes in the cells during apoptosis, images were obtained in the transmitted-light channel.

### Animal tumor model

The study was carried out in compliance with the ARRIVE guidelines. All animal protocols were approved by the Ethics Committee of the Privolzhsky Research Medical University (Russia). The experiments were performed on female Balb/c mice, 20–22 g body weight, purchased from the Pushchino animal nursery (Pushchino, Russia). Six animals were inoculated subcutaneously in the left flank with 5 × 10^5^ CT26 cells expressing mKate2-DEVD-iRFP in 100 μL phosphate buffered saline (PBS). FLIM was implemented in vivo on the 14–15th day of tumor growth when the tumors had reached 4–5 mm in diameter. Staurosporine was injected intratumorally at a dosage of 3.3 mg/kg (3.3 μg in 50 μL 13% DMSO, administered in 4–6 injections) 2 h prior to FLIM. Control mice received intratumoral injection of 50 μL of DMSO.

Before the imaging procedure, the mice were anesthetized intramuscularly with a mixture of Zoletil (40 mg/kg, 50 μL, Virbac SA, France) and 2% Rometar (10 mg/kg, 10 μL, Spofa, Czech Republic), and a skin flap over the tumor was surgically opened. Each anesthetized mouse was placed on a glass coverslip fixed in the microscope stage in such a way that the tumor was located adjacent to the objective. For each microscopic field of view the FLIM of the mKate2 and of NAD(P)H were performed sequentially. The FLIM images were captured from between 4–6 fields of view for each mouse tumor.

Once imaging was completed, the animals were sacrificed by cervical dislocation, and the tumors were excised for histopathological analysis.

### Histology

Immediately after removal, the tumors were placed in 10% neutral-buffered formalin. The formalin-fixed specimens were embedded in paraffin, sectioned into 5 μm slices and stained with hematoxylin and eosin (H&E). The tissue slides were examined using a Leica DM2500 microscope (Leica, Germany).

### Two-photon FLIM

Two-photon FLIM was performed on an LSM 880 (Carl Zeiss, Germany) laser scanning microscope equipped with a Mai Tai HP (Spectra Physics, USA) Ti:Sa femtosecond laser (repetition rate 80 MHz, pulse duration 140 fs) and an FLIM module based on a time-correlated single photon SPC-150 counting card and HPM-100-40 hybrid detector (Becker&Hickl GmbH, Germany). A C Plan-Apochromat 40×/1.3 NA oil immersion objective was used in all of the experiments.

The fluorescence intensity and lifetime data were processed with ImageJ (NIH, USA) and SPCImage software (Becker & Hickl GmbH, Germany), respectively.

#### Caspase-3 activity analysis

Caspase-3 activity was detected by measuring the mean fluorescence lifetime (τ_m_) of mKate2, the donor in the sensor mKate2-DEVD-iRFP. Fluorescence of mKate2 was excited at 1000 nm and the emission was detected in the range 595–670 nm. The FLIM image acquisition time was 60 s, resulting in ≥ 5000 photons per decay curve (binning 1). The fluorescence lifetime of mKate2 was calculated following a monoexponential fitting procedure with an average goodness of fit of χ^2^ ≤ 1.2.

#### NAD(P)H and FAD analysis

Two-photon fluorescence of NAD(P)H and FAD was excited at the wavelengths 750 nm and 900 nm, respectively, and the emissions were detected in the ranges 450–490 nm and 500–550 nm, respectively. The FLIM image acquisition time was 60 s. A binning factor of 1–2 was applied for NAD(P)H, and of 3–4 for FAD to adjust the number of photons to ≥ 5000 per decay curve. The fluorescence decay curves were fitted with a two- or three-exponential decay model. In the case of three-exponential fitting, the τ_1_ value was fixed at 0.45 ns to relax the requirements on the minimum photon numbers and to speed up the computational times. The goodness of the fit, the χ^2^ value, was ≤ 1.20. NAD(P)H and FAD were analyzed in the cytoplasm of the cells by using the ROI option.

The intensity-based redox ratio FAD/NAD(P)H was calculated from the corresponding two-photon fluorescence images after subtracting the background.

### Intracellular ROS detection

2′,7′-Dichlorodihydrofluorescein diacetate (H_2_DCFDA) (Sigma-Aldrich) was used to detect intracellular ROS. For this, H_2_DCFDA (25 μM) was added to the cells after washing with PBS and incubated for 30 min. H_2_DCFDA is oxidized to fluorescent 2′,7′-dichlorofluorescein (DCF) in the presence of ROS. In a parallel experiment, 5-(and-6)-carboxy-2′,7′-dichlorofluorescein diacetate (CDCFDA) (Sigma-Aldrich), an oxidation-insensitive dye, was used to normalize uptake, efflux, and ester cleavage of H_2_DCFDA. CDCFDA is hydrolyzed to fluorescent CDCF by intracellular esterases. CDCFDA (10 μM) was added to the cells after washing with PBS and incubated for 30 min in the dark. Fluorescence of DCF or CDCF was excited by an argon laser at a wavelength of 488 nm and registered in the range 520–550 nm. For the control, untreated cells were stained, while the rest were stained at the selected time-points after treatment. The fluorescence intensity was calculated for individual cells with ImageJ and normalized to the corresponding control value.

### Statistical analysis

The mean values (M) and standard deviations (SD) were used to express the data. The data were compared using the one-way ANOVA with Bonferroni post hoc test. P-values ≤ 0.05 were considered statistically significant. Pearson’s correlation test was applied to determine the linear regression between the parameters. The median, 25th and 75th percentiles, minimum and maximum were used to express the data of the ROS assay. The total number of cells used for the mean value calculations was from 20 to 50 in 5 fields of view.

## Supplementary Information

Supplementary Information.
