# Author Correction: Insight into redox regulation of apoptosis in cancer cells with multiparametric live-cell microscopy

**DOI:** 10.1038/s41598-022-12901-2

**Published:** 2022-05-31

**Authors:** Marina V. Shirmanova, Alena I. Gavrina, Tatiana F. Kovaleva, Varvara V. Dudenkova, Ekaterina E. Zelenova, Vladislav I. Shcheslavskiy, Artem M. Mozherov, Ludmila B. Snopova, Konstantin A. Lukyanov, Elena V. Zagaynova

**Affiliations:** 1 Privolzhsky Research Medical University, Minin and Pozharsky Sq. 10/1, 603005 Nizhny Novgorod, Russia; 2 National Medical Research Radiological Centre of the Ministry of Health of the Russian Federation, 2nd Botkinsky proezd, 3, Moscow, Russia 125284; 3 Becker&Hickl GmbH, Nunsdorfer Ring 7-9, 12277 Berlin, Germany; 4 Skolkovo Institute of Science and Technology, Bolshoy Boulevard 30, bld. 1, Moscow, Russia 121205; 5 Lobachevsky State University of Nizhny Novgorod, Gagarin Avenue 23, Nizhny Novgorod, Russia 603950

Correction to: *Scientific Reports* https://doi.org/10.1038/s41598-022-08509-1, published online 16 March 2022

The original version of this Article contained errors in the in-text citations.

In the Discussion:

“It has been suggested that cisplatin-induced ROS generation occurs as a consequence of its direct effect on mitochondrial DNA, resulting in impairment of ETC protein synthesis^21^ and enhanced mitochondrial biogenesis^24^. In H_2_O_2_-induced apoptosis, the release of mitochondrially generated ROS has also been identified^25^.”

now reads:

“It has been suggested that cisplatin-induced ROS generation occurs as a consequence of its direct effect on mitochondrial DNA, resulting in impairment of ETC protein synthesis^24^ and enhanced mitochondrial biogenesis^25^. In H_2_O_2_-induced apoptosis, the release of mitochondrially generated ROS has also been identified^26^.”

“Previously, Ricci et al. using staurosporine, actinomycin D, or UV as apoptosis inducers, suggested that the production of ROS during apoptosis can be caspase dependent^26^.”

now reads:

“Previously, Ricci et al. using staurosporine, actinomycin D, or UV as apoptosis inducers, suggested that the production of ROS during apoptosis can be caspase dependent^27^.”

“On the other hand, results by Brentnall et al. suggested that caspase-3 inhibits ROS production, while caspase-7 may be required for ROS accumulation during intrinsic apoptosis^27^.”

now reads:

“On the other hand, results by Brentnall et al. suggested that caspase-3 inhibits ROS production, while caspase-7 may be required for ROS accumulation during intrinsic apoptosis^28^.”

“In the case of H_2_O_2_-induced apoptosis, early mitochondrial events, such as the generation of permeability transition pores, a drastic decrease in the mitochondrial transmembrane potential, and the release of mitochondrial ROS, precede the activation of caspase-3^25^.”

now reads:

“In the case of H_2_O_2_-induced apoptosis, early mitochondrial events, such as the generation of permeability transition pores, a drastic decrease in the mitochondrial transmembrane potential, and the release of mitochondrial ROS, precede the activation of caspase-3^26^.”

“It has previously been verified that the intensity-based redox ratios FAD/(NADH + FAD) or FAD/NADH are highly correlated with biochemical assessments of the NAD^+^/(NADH + NAD^+^) ratio using mass spectrometry^28^ or indicated by assessing cellular oxygen consumption^29^.”

now reads:

“It has previously been verified that the intensity-based redox ratios FAD/(NADH + FAD) or FAD/NADH are highly correlated with biochemical assessments of the NAD^+^/(NADH + NAD^+^) ratio using mass spectrometry^29^ or indicated by assessing cellular oxygen consumption^30^.”

“Paralleling our study, increases in the redox ratio FAD/(NADH + FAD) and in ROS were detected by Podsednik et al. in breast cancer cells following the addition of H_2_O_2_^30^.”

now reads:

“Paralleling our study, increases in the redox ratio FAD/(NADH + FAD) and in ROS were detected by Podsednik et al. in breast cancer cells following the addition of H_2_O_2_^31^.”

“This regulatory mechanism is, at least partly, involved in both H_2_O_2_- and STS-induced apoptosis^31^ and explains our observation that caspase-3 activity correlates with NADH-a_2_ upon treatment with H_2_O_2_ or STS. In the case of cisplatin, the increase of NADH-a_2_ was a non-specific response, observed in all cells. This may also be due to increased OXPHOS, as it is considered a beneficial, adaptive response to DNA damage^32^. Previously, we have demonstrated that increased NADH-a_2_ and FAD/NADH ratios are common responses of cancer cells to chemotherapeutic drugs, including cisplatin^33^.”

now reads:

“This regulatory mechanism is, at least partly, involved in both H_2_O_2_- and STS-induced apoptosis^32^ and explains our observation that caspase-3 activity correlates with NADH-a_2_ upon treatment with H_2_O_2_ or STS. In the case of cisplatin, the increase of NADH-a_2_ was a non-specific response, observed in all cells. This may also be due to increased OXPHOS, as it is considered a beneficial, adaptive response to DNA damage^33^. Previously, we have demonstrated that increased NADH-a_2_ and FAD/NADH ratios are common responses of cancer cells to chemotherapeutic drugs, including cisplatin^34^.”

“On the other hand, NADPH plays an important role as a source for ROS being a cofactor of the NADPH oxidases (NOX)^34–40^.”

now reads:

“On the other hand, NADPH plays an important role as a source for ROS being a cofactor of the NADPH oxidases (NOX)^35–40^.”

The original Article has been corrected.

