# Supplementary material for: Insight into redox regulation of apoptosis in cancer cells with multiparametric live-cell microscopy
Source: Sci Rep. 2022 Mar 16;12:4476. doi: 10.1038/s41598-022-08509-1 (PMC8927414; doi:10.1038/s41598-022-08509-1)

**Supplementary Materials**

**Insight into redox regulation of apoptosis in cancer cells with multiparametric live-cell microscopy**

Marina V. Shirmanova^1*^, Alena I. Gavrina^1^, Tatiana F. Kovaleva^1^, Varvara V. Dudenkova^1^, Ekaterina E. Zelenova^2^, Vladislav I. Shcheslavskiy^1,3^, Artem M. Mozherov^1^, Ludmila B. Snopova^1^, Konstantin A. Lukyanov^4^, Elena V. Zagaynova^1,5^

1 - Privolzhsky Research Medical University, Minin and Pozharsky Sq. 10/1, 603005 Nizhny Novgorod, Russia

2 - National Medical Research Radiological Centre of the Ministry of Health of the Russian Federation, 2nd Botkinsky proezd, 3, 125284, Moscow, Russia

3 - Becker&Hickl GmbH, Nunsdorfer Ring 7-9, 12277 Berlin, Germany

4 - Skolkovo Institute of Science and Technology, Bolshoy Boulevard 30, bld. 1, 121205 Moscow, Russia

5 - Lobachevsky State University of Nizhny Novgorod, Gagarin Avenue 23, 603950 Nizhny Novgorod, Russia

**Table S1.** Fluorescence lifetimes of protein-bound NAD(P)H (τ_2_) in CT26 cells upon induction of apoptosis. P-values are shown only for statistically significant differences with control (0 h).


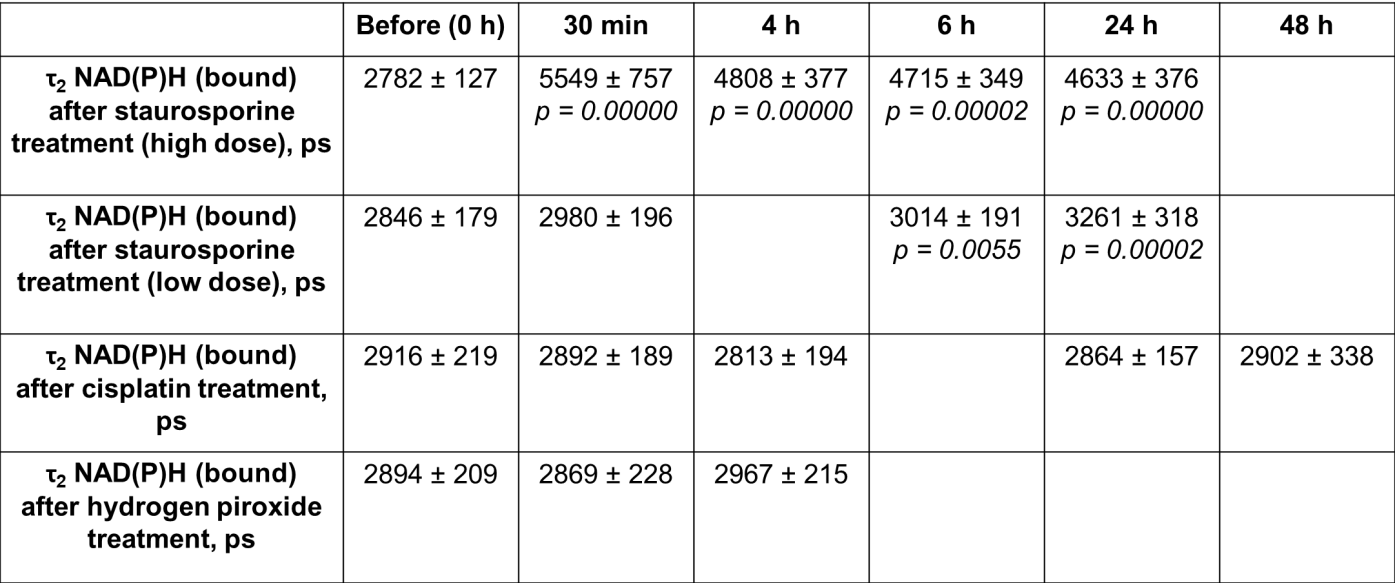


**
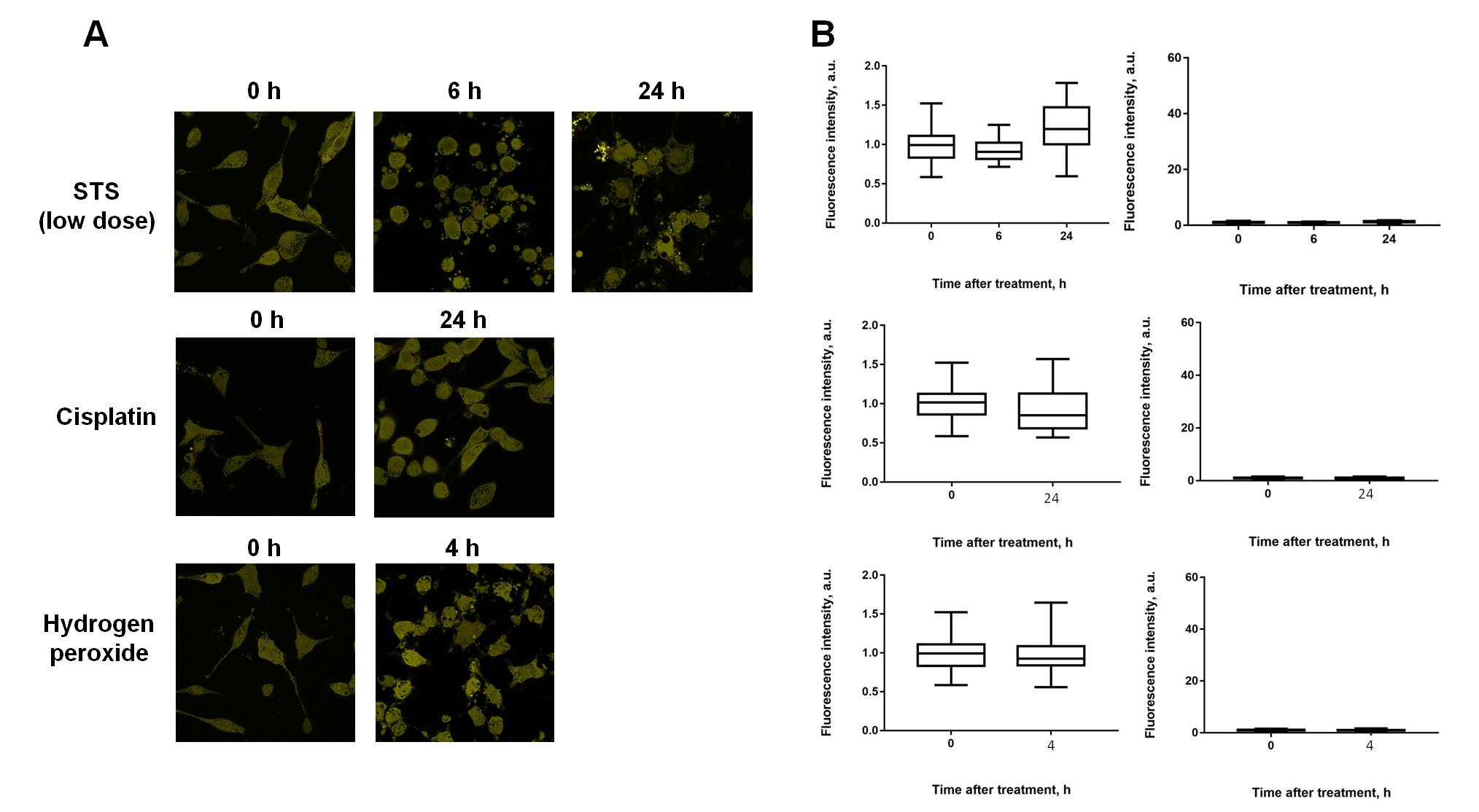
**

**Figure S1.** Control experiment using oxidation-insensitive dye CDCFDA. (A) Time-lapse microscopic images of CT26 cells stably expressing the genetically encoded sensor, mKate2-DEVD-iRFP, stained with the CDCFDA, before (0 h) and after treatment with staurosporine (STS), cisplatin or hydrogen peroxide. Image size is 213 × 213 μm. (B) Quantification of fluorescence intensity of CDCF in the cells. Box-and-Whisker plots display the median, 25^th^ and 75^th^ percentiles, minimum and maximum. n=20–30 cells. Box-and-Whisker plots are shown in two different scales.

**Table S2.** Correlations between caspase-3 activity (τ_m_ mKate2), the redox ratio FAD/NAD(P)H (RR), protein-bound NADPH fraction (a_3_ NADPH) and ROS level in cancer cells upon induction of apoptosis.


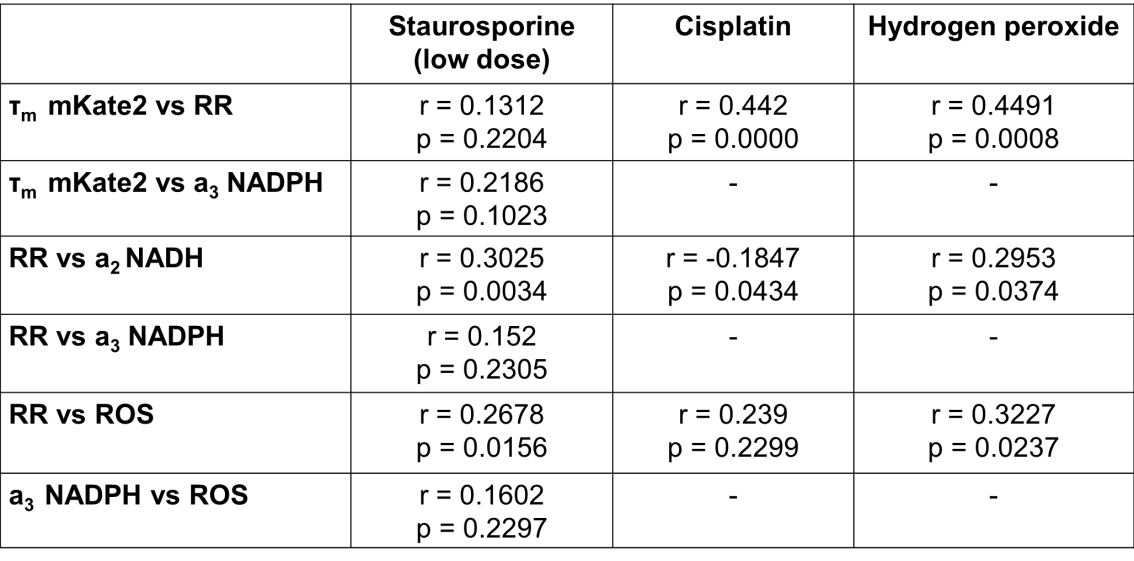

Supplement: Supplementary file 1 — Supplementary Information. [file 41598_2022_8509_MOESM1_ESM.docx]
